# Modeling Spring-In of L-Shaped Structural Profiles Pultruded at Different Pulling Speeds †

**DOI:** 10.3390/polym13162748

**Published:** 2021-08-16

**Authors:** Alexander Vedernikov, Alexander Safonov, Fausto Tucci, Pierpaolo Carlone, Iskander Akhatov

**Affiliations:** 1 Center for Design, Manufacturing and Materials, Skolkovo Institute of Science and Technology, 30/1 Bolshoi Boulevard, 121205 Moscow, Russia; a.safonov@skoltech.ru (A.S.); i.akhatov@skoltech.ru (I.A.); 2 Department of Industrial Engineering, University of Salerno, Via Giovanni Paolo II, 132-84084 Fisciano, Italy; ftucci@unisa.it (F.T.); pcarlone@unisa.it (P.C.)

**Keywords:** pultrusion, spring-in, finite element analysis (FEA), cure behavior, process modeling

## Abstract

Cure-induced deformations are inevitable in pultruded composite profiles due to the peculiarities of the pultrusion process and usually require the use of costly shimming operations at the assembly stage for their compensation. Residual stresses formed at the production and assembly stages impair the mechanical performance of pultruded elements. A numerical technique that would allow the prediction and reduction of cure-induced deformations is essential for the optimization of the pultrusion process. This study is aimed at the development of a numerical model that is able to predict spring-in in pultruded L-shaped profiles. The model was developed in the ABAQUS software suite with user subroutines UMAT, FILM, USDFLD, HETVAL, and UEXPAN. The authors used the 2D approach to describe the thermochemical and mechanical behavior via the modified Cure Hardening Instantaneous Linear Elastic (CHILE) model. The developed model was validated in two experiments conducted with a 6-month interval using glass fiber/vinyl ester resin L-shaped profiles manufactured at pulling speeds of 200, 400, and 600 mm/min. Spring-in predictions obtained with the proposed numerical model fall within the experimental data range. The validated model has allowed authors to establish that the increase in spring-in values observed at higher pulling speeds can be attributed to a higher fraction of uncured material in the composite exiting the die block and the subsequent increase in chemical shrinkage that occurs under unconstrained conditions. This study is the first one to isolate and evaluate the contributions of thermal and chemical shrinkage into spring-in evolution in pultruded profiles. Based on this model, the authors demonstrate the possibility of achieving the same level of spring-in at increased pulling speeds from 200 to 900 mm/min, either by using a post-die cooling tool or by reducing the chemical shrinkage of the resin. The study provides insight into the factors significantly affecting the spring-in, and it analyzes the methods of spring-in reduction that can be used by scholars to minimize the spring-in in the pultrusion process.

## 1. Introduction

Pultrusion is the most efficient process for producing composite structural profiles of constant cross-sections [1,2,3]. Owing to their high strength-to-weight ratio [4,5], superior corrosion resistance [6,7], and improved durability [8,9], pultruded profiles have been successfully used as structural elements in the fields of bridge construction [10,11,12], civil [13,14], and architectural engineering [15,16]; marine construction [17,18]; aerospace and aviation engineering [19,20,21]; transportation [22,23]; and energy systems [24]. However, process-induced deformations, such as spring-in (common in curved elements) and warpage (common in flat elements), may result in a certain loss in the economic efficiency of mass production of composite profiles [25]. Spring-in is the primary contributor [26] to the distortion of a profile, which may require costly and time-consuming shimming operations during assembly [27,28]. Residual stresses developing during the production and assembly stages are detrimental to the final mechanical performance of a structure [29,30]. Thus, the ability to predict, control, and compensate for process-induced deformations is crucial for the effective design and assembly of composite structures [31,32].

The phenomenon of spring-in can be observed in various processes of composite manufacturing, such as autoclave [33,34,35,36], resin transfer molding [37,38,39], vacuum-assisted resin transfer molding [40,41], vacuum bagging [42,43], compression molding [44,45], filament winding [46], microwave curing [47], and pultrusion [48,49]. The main causes of spring-in are the anisotropy of the mechanical properties of a composite [50], [51,52]; chemical [53,54] and thermal [55,56] shrinkage of a material; nonuniform distribution of temperatures [57]; and curing degree [58,59]. The peculiarities of the pultrusion process also contribute to the spring-in because a composite material undergoes all process stages sequentially, i.e., impregnation, heating, polymerization, and cooling. Therefore, the structural properties of the produced profiles depend upon the process conditions used in production [60]. Hence, the creation of numerical models predicting the influence of process conditions on the value of spring-in is a vital problem for the pultrusion process optimization [61,62,63].

Successful simulation of the pultrusion process requires a model that describes the distribution of temperatures in a composite along with the matrix polymerization process [64,65,66,67] and mechanical behavior of a composite during manufacturing [68,69]. To model residual stresses and distortions, Baran et al. [49] applied a 3D approach to solve the thermochemical problem and a 2D approach to explain the mechanical behavior via the Cure Hardening Instantaneous Linear Elastic (CHILE) model. In a follow-up study, they showed that the spring-in value depends on the pulling speed [48]. Wang et al. [70] proposed the numerical model to predict the spring-in and conducted an experiment to compare predictions and experimental results. Predictions were found to be in good agreement with experimental data. It was also found that the contribution of chemical shrinkage into deformations is significantly higher compared to that of thermal expansion. However, those contributions were not quantified.

Despite the lack of studies on spring-in formation in pultruded composites, over the last 30 years, a large number of experimental and numerical studies on the subject have been published in relation to other composite manufacturing processes. Those works studied the influence of cure cycle schedule, thermal shrinkage, and chemical shrinkage on the evolution of cure-induced residual stresses and deformations. Back in 1992, Bogetti and Gillespie [57] proposed a model that is capable of predicting the mechanical characteristics, thermal and chemical strains in resin during polymerization. The study demonstrated the major role of thermal shrinkage and chemical shrinkage in the development of residual stresses and deformations. Jain and Mai [71] have proposed a model based on the modified shell theory, which predicted the evolution of residual stresses and deformations such as spring-in. They have shown that chemical shrinkage, among other factors, has a significant effect on the evolution of residual stresses and shape distortions. Wiersma et al. [53], aiming to build the model capable of accurate prediction of spring-in in L-shaped composites, have considered the thermoelastic model accounting for thermal shrinkage, and the viscoelastic model accounting for irreversible effects occurring during resin polymerization (chemical shrinkage and viscosity evolutions). Subsequently, Radford and Rennick [51] have quantified the contribution of thermoelastic and non-thermoelastic components in spring-in distortion of carbon fiber/epoxy brackets manufactured by the autoclave technique. In 2001, Svanberg and Holmberg [37] studied the influence of the cure cycle on spring-in evolutions in the resin transfer molding process. They distinguished three major factors accounting for spring-in, i.e., thermal shrinkage, chemical shrinkage, and frozen-in deformations. While studying the cure quenching phenomenon, Ersoy et al. [72] were able to isolate the contribution of thermal shrinkage (happening before and after vitrification) and of chemical shrinkage to spring-in formation. In 2006, Ruiz and Trochu [73] demonstrated the methodology allowing a researcher to optimize polymerization in a liquid composite molding process by way of minimizing the residual stresses resulting from chemical and thermal shrinkage. The methodology makes it possible to improve the process of resin curing while simultaneously minimizing the process time and residual stresses. In the quenching experiment, Wisnom et al. [50] have shown the development of spring-in at different stages of the cure cycle—initial growth, peak, and reduction during subsequent polymerization. It was shown that at the rubbery state, both thermal and chemical shrinkage affect spring-in evolutions, with the contribution of chemical shrinkage constituting as much as 50%. In Wisnom et al. [27], the authors proposed and verified experimentally the analytical solution describing the mechanism of spring-in formation due to thermal and chemical shrinkage, taking place between gelation and vitrification of the resin. Hsiao and Gangireddy [41] in 2008 used the vacuum-assisted resin transfer molding (VARTM) process to prove experimentally that the addition of 1.5 wt % carbon nanofibers to polyester resin allows a spring-in reduction by as much as 73% through a reduction of deformations caused by thermal and chemical shrinkage. The analytical solution and the 3D FEA model proposed confirm the experimental results and predict a complete elimination of spring-in at 10 wt % of carbon nanofibers. Li et al. [47] have shown that it is possible to significantly reduce the cure-induced strains in carbon fiber-reinforced bismaleimide composites by replacing conventional thermal curing process for the microwave one and to achieve spring-in reduction in the L-shaped structure by as much as 1.2°. Subsequently, Kravchenko et al. [74] conducted the experimental and numerical study of deflection in bi-lamina strips caused by thermal and chemical shrinkage, occurring at various stages of the cure cycle. Takagaki et al. [75] in 2017 used Fiber Bragg Grating (FBG) sensors to experimentally measure through-thickness normal and shear strains. The results obtained were used to analyze the curing process and the development of spring-in in the L-shaped carbon-fiber-reinforced polymer (CFRP) part at different stages of the cure process, which are associated with chemical and thermal shrinkage. Nawab et al. [76] showed numerically the evolution of spring-in in a carbon/epoxy woven composite bracket at different stages of the cure cycle and subsequent cooling, and they established that the contribution of thermal shrinkage constituted 81%, while that of chemical shrinkage during curing constituted only 19%. Hu et al. [77] demonstrated an in situ method to monitor gelation and determine the evolution of effective chemical shrinkage during polymerization, using the FBG sensors. The authors have manufactured the C-shape composite specimens and compared the spring-in values predicted with the use of the thermal model (accounting only for the thermal shrinkage) and of Rennick’s model [51]. Three methods were used to measure the chemical shrinkage—by bi-material strip, by Thermal Mechanical Analysis, and by FBG sensors—with the latter producing the most reliable results. Exner et al. [78] have shown experimentally that the addition of aluminum oxide nanoparticles in the amount of at least 5 wt % reduces chemical and thermal shrinkage, resulting in a reduction of spring-in in vacuum-infused CFRP L-shaped composites. In 2019, Groh et al. [79] in the experimental study of RTM-fabricated L-shaped CFRP composites based on Fast Curing Epoxy Resin have shown the absence of relation between the spring-in and the cooling rate. Subsequently, Qiao and Yao [80] proposed the 3D numerical model and calculated the contributions of thermal shrinkage, chemical shrinkage, and of tool–part interaction to the spring-in in the L-shaped structure. It was found that the contribution of thermal shrinkage is almost independent of part thickness, and that of the chemical shrinkage reduces with increase in part thickness. It was also found that the spring-in caused by chemical shrinkage is higher compared to that caused by thermal shrinkage. Shaker et al. [81] through the addition of 5% of silica microparticle fillers were able to reduce the coefficient of thermal expansion of resin and, as a result, to reduce the spring-in in glass/vinyl ester L-shaped composite parts by as much as 65%, from 1.807° to 0.632°. Recently, Struzziero et al. [82] conducted the experimental and numerical study of the residual stress and warpage deformations during cure in laminates produced by VARTM. The authors used the multi-objective optimization and proposed the method of reducing the warpage by as much as 10% and manufacturing time by 33%.

The majority of studies on the subject discussed here are devoted to the composite manufacturing processes other than pultrusion. Hence, the insights from those studies cannot be fully applied when studying the pultrusion process, due to the unique features of pultrusion as the manufacturing process. However, the few existing studies of spring-in in pultruded composites fail to explore the subject comprehensively. For example, these studies do not provide experimental validation of numerical models at various pulling speeds. Moreover, no thorough analysis has been conducted on the reasons for spring-in increase at higher pulling speeds. In addition, the authors failed to separate and evaluate the contributions from thermal and chemical shrinkage to the final value of spring-in during the polymerization and cooling phases. This study is aimed at the analysis of thermal and chemical shrinkage influence on the evolutions of spring-in in L-shaped profiles taking place at different pulling speeds. This study also aims to investigate the ways to minimize the spring-in through the use of a post-die cooling tool or by reducing the chemical shrinkage of the resin. The outcomes of this study can be used by researchers to minimize spring-in deformations occurring during pultrusion.

This paper presents a numerical and experimental study of the influence of pulling speed on the value of spring-in in L-shaped structural pultruded profiles of 75 mm × 75 mm × 6 mm. The pultrusion of vinyl ester-based profiles reinforced with unidirectional glass fiber rovings and fabrics was carried out. Two pultrusion experiments were performed, with a 6-month interval. The spring-in in pultruded profiles was measured on the same day of manufacture after the profiles got cooled to room temperature. A numerical simulation of the pultrusion process at different pulling speeds was performed using a 2D approach to thermochemical and mechanical behavior via the modified CHILE model. The results demonstrate good correlation between numerical predictions and experimental values of spring-in. The study also evaluated the contribution of each mechanism to the formation of spring-in. The results show that the largest contribution to spring-in comes from the chemical shrinkage of the matrix after the exit from the die block and the thermal shrinkage of the composite when cooling to the glass transition temperature. Numerical simulation results were used to analyze the possibility of reducing the spring-in with the help of a post-die cooling tool or by reducing the chemical shrinkage of the resin.

## 2. Materials and Methods

### 2.1. Pultrusion Manufacturing

**Figure 1:**
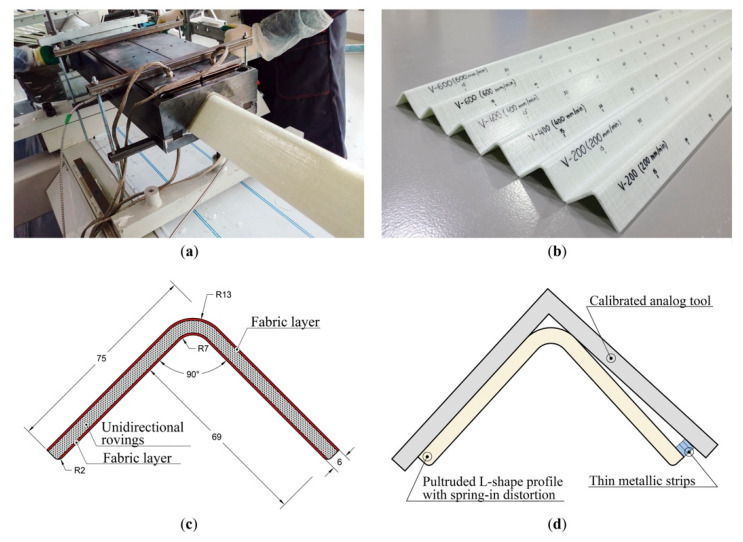
Pultrusion process setup: (**a**) Pultrusion of an L-shaped profile; (**b**) L-shaped profiles pultruded at pulling speeds of 200, 400, and 600 mm/min; (**c**) The cross-section of a 75 mm × 75 mm × 6 mm L-shaped pultruded profile and the position of the unidirectional rovings and fabric layers; (**d**) Schematic representation of the spring-in measurement process.

The profiles used for the experiments were manufactured using the Pultrex Px500-6T pultrusion machine (Pultrex, Lawford, UK) at the Laboratory of Composite Materials and Structures of the Center for Design, Manufacturing and Materials (Skolkovo Institute of Science and Technology, Moscow, Russia) (Figure 1a). Two pultrusion experiments with 75 mm × 75 mm × 6 mm L-shaped structural profiles (Figure 1c) have been conducted with a 6-month interval. In total, 104 threads of E-glass unidirectional rovings PS 2100 (Owens Corning Composite Materials, Toledo, OH, USA) with a linear density of 9600 TEX (9600 g/1000 m), and two layers of E-glass fabric LT 0600/S 300/06H 01/125 GUS (Owens Corning Composite Materials, Toledo, OH, USA) with a surface density of 900 g/m^2^ were utilized as reinforcement. The matrix was composed of Atlac 430 vinyl ester resin (DSM Composite Resins AG, Schaffhausen, Switzerland) with the following additives: Triganox C (Akzo Nobel Polymer Chemicals B.V., Amsterdam, The Netherlands), Perkadox 16 (Akzo Nobel Polymer Chemicals B.V., Amsterdam, The Netherlands), BYK-A555 (BYK Additives & Instruments, Wesel, Germany), and zinc stearate (Baerlocher GmbH, Unterschleißheim, Germany). To fabricate the profiles, the 600 mm-long steel die block was used, with four 350 mm-long heating platens installed by pairs at the top and the bottom of the die block along the pulling axis. In order to control the die block temperature, two thermocouples were installed within the body of the die block. The allowable temperature range was 145 ± 10 °C. In total, six 1.5 m-long profiles were manufactured in two experiments at pulling speeds of 200, 400, and 600 mm/min (see Figure 1b). The spring-in angle was measured 3 h after the pultrusion experiment, after the profiles had cooled to the ambient temperature. To measure the spring-in, a set of thin metallic strips (thicknesses of 0.1–1 mm) and a calibrated L-shaped right-angled tool to ensure the correctness of 90° angle measurements were used [83]. The required number of metallic strips were placed in the gap between the leg of the profile and angle tool, and the total thickness of the strips ($t_{s}$) was registered (Figure 1d). The angular value of spring-in (φ) is determined based on the size of the profile legs ($L_{w}$ = 62 mm) and the measured total thickness of metallic strips inserted into the gap ($t_{s}$) [60]:

(1) $$\varphi =\frac{180{}^{\circ}}{\pi }\arctan\left(t_{s}/L_{w}\right).$$

The accuracy of this spring-in measurement method constituted ±0.09°. The average of all values measured at several sections along the length of the profile was taken as the final value of spring-in [84].

### 2.2. Modeling

In this section, a thermomechanical initial-boundary value problem (IBVP) is discussed. A numerical modeling tool is used to predict the response of a body to applied temperature loads. This can be achieved by solving the given IBVP problem. A more detailed description of this IBVP statement can be found in Zocher et al. [85] and in Svanberg and Holmberg [56]. Two problems should be solved in resin polymerization modeling, namely, the heat transfer problem (Section 2.2.1) and the mechanical problem (Section 2.2.2). As the properties of resin and, therefore, of composite material depend on the temperature, in order to solve this IBVP, the distribution of temperature and polymerization degree was modeled with Equations (2)–(8) of the 2D thermochemical model presented in Section 2.2.1. Then, based on results obtained and using the equations presented in Section 2.2.2, it was possible to determine the cure- and temperature-dependent Young’s modulus (Equations (9) and (10)), bulk modulus (Equations (11) and (12)), and Poisson’s ratio (Equation (13)) of the resin. The given mechanical properties of the resin were used further to calculate mechanical properties of a composite, based on the Self-Consistent Field Micromechanics (SCFM) approach [86,87]. Finally, the obtained mechanical properties of a composite are used to determine the stress–strain state in the composite. A more detailed description of the mechanical problem statement of process-induced residual stresses and distortions can be found in Baran et al. [49]. The novelty of the mechanical problem presented here is in the more accurate CHILE model that uses seven regions to describe changes in Young’s modulus of resin, and it accounts for changes in Poisson’s ratio of the matrix during phase transitions.

Earlier, it was shown that axial conduction can be neglected when solving the temperature problem [88]. In addition, no significant differences were found in the distributions of stresses and displacements in the transverse direction, which were obtained in 2D and 3D simulations of mechanical behavior [89]. Thus, to accelerate computations, a two-dimensional model was used to solve the thermochemical and mechanical problems in this study. However, these assumptions may result in overestimated values of thermal peak. In addition, the 2D approach makes it impossible to account for stresses, occurring along the longitudinal axis of the profile and causing additional shape distortions in the pulling direction. Nevertheless, further, it will be shown that these assumptions are reasonable and will not result in large discrepancy between predicted and experimental spring-in data.

#### 2.2.1. 2D Thermal Model

A steady-state pultrusion process with a pulling speed of u is considered. By disregarding the heat conduction along the length of the composite profile, the heat transfer equation in a Lagrangian (material) frame of reference can be expressed as follows [88]:

(2) $$C_{p\_\mathrm{comp}}\left(T\right){\;\rho }_{\mathrm{comp}}\frac{\partial T}{\partial t}=k_{\mathrm{comp}}\frac{\partial^{2}T}{\partial x^{2}}+k_{\mathrm{comp}}\frac{\partial^{2}T}{\partial y^{2}}+q,$$

where x and y are the coordinates of a cross-section of the composite profile, T is instantaneous temperature, $C_{p\_\mathrm{comp}}\left(T\right)$ is the temperature-dependent heat capacity of a composite material, $\rho_{\mathrm{comp}}$ is the composite density, $k_{\mathrm{comp}}$ is the thermal conductivity of the composite in the cross-sectional plane, and q is the heat released due to the exothermic reaction in a polymer matrix. As the heat equation is expressed in the Lagrangian (material) frame of reference, the pulling speed (u), absent in Equation (2), affects the boundary condition equations (Equations (3) and (4)), corresponding to the position of the composite profile cross-section inside or outside the die, respectively:

(3) $$k_{\mathrm{comp}}{\frac{\partial T}{\partial n}|}_{\Gamma }=-h_{\mathrm{die}}\left(T-T_{\mathrm{die}}\left(z\right)\right)\;\mathrm{at}\;z=\mathrm{ut}<L_{\mathrm{die}},$$

(4) $$k_{\mathrm{comp}}{\frac{\partial T}{\partial n}|}_{\Gamma }=-h_{\mathrm{air}}\left(T-T_{\mathrm{amb}}\left(z\right)\right)\;\mathrm{at}\;z=\mathrm{ut}\geq L_{\mathrm{die}},$$

where Γ is the surface of the profile, $h_{\mathrm{die}}$ is the coefficient of convective heat transfer between the die block and the profile, $h_{\mathrm{air}}$ is the coefficient of convective heat transfer between the air and the profile after exiting the die block, $T_{\mathrm{die}}$ is the temperature of the die block, changing along the pulling direction z, and $T_{\mathrm{amb}}$ is the ambient temperature.

Assuming the temperature of the composite at the die block entrance, $T_{\mathrm{in}}$, to be uniform over the entire cross-section, it can be expressed as follows:

(5) $${T|}_{t=0}=T_{\mathrm{in}},$$

The internal heat released due to the exothermic reaction of the resin (q) during polymerization can be expressed as:

(6) $$q=\left(1-V_{f}\right)\rho_{R}H_{\mathrm{tot}}\frac{d\alpha }{\mathrm{dt}},$$

where $V_{f}$ is the volume fraction of reinforcement in a composite, $\rho_{r}$ is the resin density, $H_{\mathrm{tot}}$ is total heat released during curing, and $\frac{d\alpha }{\mathrm{dt}}$ is the resin curing rate.

To describe the rate of resin polymerization the equation of the n-th order, an autocatalytic reaction is used [90]:

(7) $$\frac{d\alpha }{\mathrm{dt}}=A_{0}e^{-\frac{E_{a}}{R\left(T+273.15\right)}}{\left(1-\alpha \right)}^{n}\left(1+K_{\mathrm{cat}}\alpha \right),$$

where $A_{0}$ is the pre-exponential coefficient, $E_{a}$ is the activation energy, R is the universal gas constant, T is the instantaneous temperature of the resin in degrees Celsius, n is the order of reaction, and $K_{\mathrm{cat}}$ is the activation constant.

It is assumed that preheating the material before the entrance into the die block will not result in its polymerization; hence, the degree of polymerization at the die block entrance is taken to be zero:

(8) $${\alpha |}_{t=0}=0.$$

#### 2.2.2. 2D Mechanical Model

It is assumed that resin starts gaining in Young’s modulus ($E_{r}$) and becomes able to sustain stresses after the gelation point ($\alpha_{\mathrm{gel}}$=0.6). To account for changes in the Young’s modulus of the resin ($E_{r}$) during the polymerization process and to describe the mechanical behavior of the resin, the CHILE model is used in its modified form [91]:

(9) $$E_{r}=\left\{ \begin{array}{cc} E_{r}^{0}, & T^{*}\leq T_{C1}\\ E_{r}^{0}+\frac{T^{*}-T_{C1}}{T_{C2}-T_{C1}}\left(E_{r}^{1}-E_{r}^{0}\right),\; & T_{C1}<T^{*}<T_{C2}\\ E_{r}^{1}+\frac{T^{*}-T_{C2}}{T_{C3}-T_{C2}}\left(E_{r}^{2}-E_{r}^{1}\right),\; & T_{C2}<T^{*}<T_{C3}\\ E_{r}^{2}+\frac{T^{*}-T_{C3}}{T_{C4}-T_{C3}}\left(E_{r}^{3}-E_{r}^{2}\right),\; & T_{C3}<T^{*}<T_{C4}\\ E_{r}^{3}+\frac{T^{*}-T_{C4}}{T_{C5}-T_{C4}}\left(E_{r}^{4}-E_{r}^{3}\right), & T_{C4}<T^{*}<T_{C5}\\ E_{r}^{4}+\frac{T^{*}-T_{C5}}{T_{C6}-T_{C5}}\left(E_{r}^{\infty }-E_{r}^{4}\right),\; & T_{C5}<T^{*}<T_{C6}\\ E_{r}^{\infty },\; & T_{C6}\leq T^{*} \end{array}\right.$$

where $T^{*}=T_{g}\left(\alpha \right)-T$ is the difference between the instantaneous glass transition temperature ($T_{g}$) and the instantaneous temperature (T) of a resin in degrees Celsius; $T_{C1}$, $T_{C2}$, $T_{C3}$, $T_{C4}$, $T_{C5}$, and $T_{C6}$ are the critical temperatures in degrees Celsius, and $E_{r}^{0}$, $E_{r}^{1}$, $E_{r}^{2}$, $E_{r}^{3}$, $E_{r}^{4}$, and $E_{r}^{\infty }$ are the corresponding elastic moduli. $T_{g}\left(\alpha \right)$ is the glass transition temperature depending on the degree of cure, which is expressed as follows [92,93]:

(10) $$T_{g}\left(\alpha \right)=T_{g0}+\left(T_{g\infty }-T_{g0}\right)\frac{\lambda \alpha }{1-\left(1-\lambda \right)\alpha },$$

where $T_{g0}$ is the glass transition temperature of the uncured resin (α=0), $T_{g\infty }$ is that of the fully cured resin (α=1), and λ is the material parameter.

To account for the changes in Poisson’s ratio during phase transitions, it should be noted that the bulk modulus of resin has the same order of magnitude in both rubber-like and glassy states [94]. According to Svanberg [56], the bulk modulus of the matrix decreases 2.5 times during the transition from the glassy ($K_{r}^{\infty }$) to the rubber-like state ($K_{r}^{0}$). By determining the bulk modulus of the matrix in glassy and rubber-like states based on the linear elastic theory (Equation (11)) [94], the instantaneous bulk modulus of the matrix ($K_{r}$) and the corresponding Poisson’s ratio ($\nu_{r}$) can be determined using Equations (12) and (13), accordingly:

(11) $$K_{r}^{\infty }=\frac{E_{r}^{\infty }}{3\left(1-2\nu_{r}^{\infty }\right)},$$

(12) $$K_{r}\left(E_{r}\right)=K_{r}^{0}+\left(K_{r}^{\infty }-K_{r}^{0}\right)\frac{E_{r}-E_{r}^{0}}{E_{r}^{\infty }-E_{r}^{0}},$$

(13) $$\nu_{r}\left(E_{r}\right)=\frac{3K_{r}-E_{r}}{6K_{r}}.$$

Then, the instantaneous mechanical properties of the resin are used to compute effective mechanical properties of the composite, using the Self-Consistent Field Micromechanics (SCFM) approach [86,87]. Thus, the obtained effective mechanical properties of a composite are subsequently applied to determine the stress–strain state in the composite [49]. The analytical relationships used to predict the effective mechanical properties and stress–strain state in the composite are presented in Supplementary Materials.

#### 2.2.3. Finite Element Modeling

The IBVP described earlier is solved by means of finite element analysis in ABAQUS FEA software suite (6.14, Dassault Systèmes SE, Vélizy-Villacoublay, France) [95]. Displacements and stresses are computed using the incremental linear elastic approach [49]. The following subroutines are used in the simulations: UMAT, FILM, USDFLD, HETVAL, and UEXPAN. The UMAT subroutine is used to calculate thermal and chemical deformations; to compute the mechanical properties of a composite, using the Self-Consistent Field Micromechanics (SCFM) approach; and to describe constitutive mechanical behavior of a composite. The FILM subroutine is used to assign temperature loads and to describe convective heat transfer between the composite and an environment, both inside and outside the die block. The USDFLD is used to define the cure degree at each point of material as a function of time and temperature; HETVAL is used to specify internal heat generation due to exothermic reaction in a polymer matrix during heat transfer analysis; and UEXPAN is used to add non-mechanical strains (thermal and chemical) to mechanical ones to obtain the total strain tensor. In order to build the model, the 4-node plane strain thermally coupled quadrilateral CPE4RT type elements are used. As the profile section is symmetric, only half of the model, consisting of 1056 elements, is used in simulations. Simulations were performed with different numbers of finite elements in order to calculate the spring-in, using the model described in Section 2.2. It was noted that increasing the number of finite elements (starting from 1056 elements), while significantly increasing the simulation time, did not lead to noticeable differences in the final value of spring-in. That is, the refinement of the mesh does not provide significant changes in simulation results.

**Figure 2:**
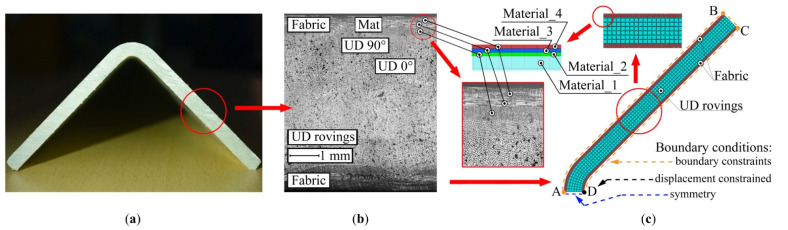
Building the numerical model of a pultruded L-shaped profile: (**a**) Cross-section of the L-shaped pultruded profile; (**b**) Microphotograph of the cross-section of the pultruded profile showing the arrangement of UD rovings and fabric layers; (**c**) Numerical model of the L-shaped pultruded profile built-in ABAQUS software.

The numerical model developed for this study uses four different material types (Figure 2c) corresponding to different types of reinforcement used in the pultrusion of L-shaped profiles (Figure 2a,b) as follows: (1) Material_1, transversely isotropic, with the axis of anisotropy oriented along the pulling direction to model the roving; (2) Material_2, transversely isotropic, with the axis of anisotropy oriented along the pulling direction to model the internal layer of fabric; (3) Material_3, with the axis of anisotropy lying within the cross-section plane and oriented parallel to the leg of the L-shaped profile to model the core layer of fabric; and (4) Material_4, transversely isotropic with axis of anisotropy oriented perpendicular to the lay-up plane to model the mat glued to the fabric. Material_1 represents the unidirectional reinforcement used to fabricate L-shaped profiles. Three materials were utilized to model the fabric material used to fabricate L-shaped profiles: Material_2, Material_3, and Material_4. In ABAQUS, Material_2 and Material_3 (representing the unidirectional reinforcement) were assigned the same material properties but with different reinforcement orientation along the direction of pultrusion and in the cross-sectional plane, respectively. Material_4 represents the material with randomly oriented reinforcement in the lay-up plane. 

For the AD edge, the symmetrical boundary conditions are set (blue dashed line in Figure 2c). The boundary constraints used to simulate the internal surface of the die block prohibit any motion at the outer perimeter of the profile inside the die block region (the ABCD segment, orange dashed line in Figure 2c). The boundary constraints are deactivated after the die block exit. In addition, any displacements of a composite at the point D are constrained (orange dashed line in Figure 2c).

**Figure 3:**
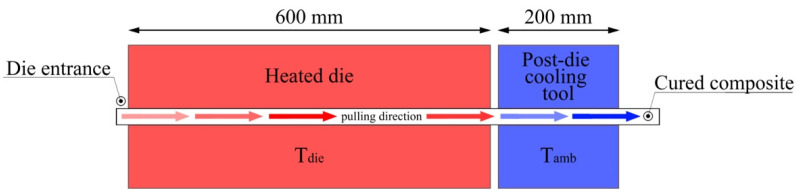
Geometry and position of the heated die and post-die cooling tool.

Furthermore, the spring-in reduction method that provides for the use of a rigid post-die cooling tool was simulated. The length of the post-die cooling tool constitutes 1/3 of the length of the die block ($L_{\mathrm{die}}/3$). It is assumed that the post-die cooling tool has constant temperature equal to the ambient temperature ($T_{\mathrm{amb}}$) and is installed immediately after the end of the heated die block. The geometry and positions of the heated die block and post-die cooling tool are shown in Figure 3.

### 2.3. Experimental Methods to Determine Model Parameters

To determine the parameters of the model, a series of thermomechanical and thermophysical tests were conducted. Test specimens were cut from plates of cured vinyl ester resin. The plates were polymerized in a laboratory vacuum drying oven XF050 (France Etuves, Chelles, France) under the following procedure: 1.5 h at 120 °C, 30 min at 150 °C, followed by natural cooling for 12 h. An Shtalmark M1-912 M/2 (Rusintermash Ltd., Pushkino, Russia) CNC milling machine was used to cut specimens from polymerized plates. The glass transition temperature and temperature dependence of the storage and loss moduli of cured resin were determined using Dynamic Mechanical Analysis (DMA) following the ISO 6721-1:2011 procedure [96], in the 3-point bending mode, with a Q800 DMA analyzer (TA Instruments Inc., New Castle, DE, USA). Measurements were taken in the temperature range of 30–170 °C, with a heating ramp of 5 °C/min, an oscillation frequency of 1 Hz, and an amplitude of 60 µm. A DSC 204 differential scanning calorimeter (NETZSCH-Gerätebau GmbH, Selb, Germany) was used to measure the heat capacity of the cured resin. Measurements were taken following the procedure specified in ISO 11357-4:2005 [97], within the temperature range of 20–100 °C, with a heating ramp of 10 °C/min. The thermal conductivity of the cured resin was measured according to the ISO 22007-4:2008 procedure [98] in the temperature range of 20–100 °C, using an LFA 457 MicroFlash laser flash apparatus (NETZSCH-Gerätebau GmbH, Selb, Germany). To determine the coefficient of thermal expansion (CTE) of cured resin, a TMA 402F thermomechanical analyzer (NETZSCH-Gerätebau GmbH, Selb, Germany) was used, following the ISO 11359-2:1999 procedure [99], at a temperature of 20 °C. The density of the cured resin was determined by hydrostatic weighing of four samples of 25 mm × 25 mm × 2 mm, using HTR-220CE electronic laboratory scales (Shinko Vibra, Tokyo, Japan).

## 3. Results

### 3.1. Model Parameters

**Figure 4:**
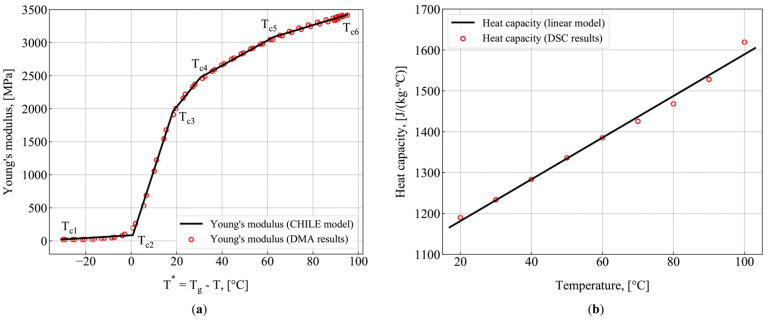
Model parameters measurements: (**a**) Young’s modulus (DMA measurements vs. CHILE model predictions); (**b**) Heat capacity (DSC measurements vs. predictions obtained with the linear approximation).

Figure 4 shows the determined parameters of the model: Young’s modulus of resin (Figure 4a) and heat capacity of resin (Figure 4b). The least squares method was used to determine the temperature-dependent specific heat and the constants of the modified CHILE model, based on experimental data. In addition, based on DMA data, the value of $T_{g\infty }$ was found, constituting $T_{g\infty }$ = 120.4 °C. The heat capacity demonstrates linear temperature dependence of the form $C_{p\_r}\left(T\right)=\left(5.1\cdot T+1080\;\right)$ J/(kg·°C). Thermal conductivity measurements conducted within the range of 20–100 °C show that the difference in thermal conductivity values does not exceed 2%. That is why thermal conductivity is assumed to be constant and equal to the average experimental value of $k_{r}$ = 0.178 W/(m·°C). The coefficient of thermal expansion measured at the temperature of 20 °C constituted $\alpha_{r}^{\infty }$ = 60·× 10^−6^ °C^−1^. The density of resin constitutes $\rho_{r}$ = 1140 kg/m^3^. All measured parameters of the model are given in Table A1.

### 3.2. Finite Element Modeling Results

The spring-in values were obtained by simulating the pultrusion of the L-shaped profile at pulling speeds of 200, 400, and 600 mm/min. The results were compared with the values obtained during the two pultrusion experiments. Then, numerical simulations were conducted to analyze the influence of pulling speed increase and of changes in Poisson’s ratio during phase transitions (according to Equation (13)) on the value of spring-in. Furthermore, the efficiency of methods of spring-in reduction was analyzed by using a post-die cooling tool or by reducing the chemical shrinkage of the matrix.

Table A1 lists the model parameters used in the computations, together with the information on the source of the data. A key feature of the model discussed here is that it uses experimentally determined values of density, temperature-dependent heat capacity, thermal conductivity, CTE, and mechanical properties of the resin (see Section 2). The values of density, heat capacity, and thermal conductivity of glass-fiber reinforcement were taken from [49], the mechanical properties of glass-fiber reinforcement were taken from [57], the kinetic constants of resin polymerization were taken from [90], and resin properties were taken from [49,56,100]. To determine the properties of the composite layers (Material_1, Material_2, Material_3, Material_4), the following analytical relationships were used: the density and heat capacity were determined as described in [88]; thermal conductivity were determined as described in [101]; the mechanical properties of Material_1, Material_2, and Material_3 were determined as described in [57]; and those of Material_4 were determined as described in [102]. The mentioned relationships and data for each Material_1, Material_2, Material_3, and Material_4 can be found in the Supplementary Materials.

In addition, at the outer perimeter of the profile, the boundary conditions of thermal contact with the ambient air, based on the given coefficient of convective heat transfer, were imposed. For the profile inside the die block, very high values of the convective heat transfer coefficient ($h_{\mathrm{die}}$ = 5000 W/(m^2^·°C)) were assigned to simulate perfect thermal contact with the die block. To simulate the thermal contact of the profile with the ambient air after the die block exit, the convective heat transfer coefficient of $h_{\mathrm{air}}$ = 9 W/(m^2^·°C) and the ambient temperature of $T_{\mathrm{amb}}$ = 18 °C were assigned.

**Figure 5:**
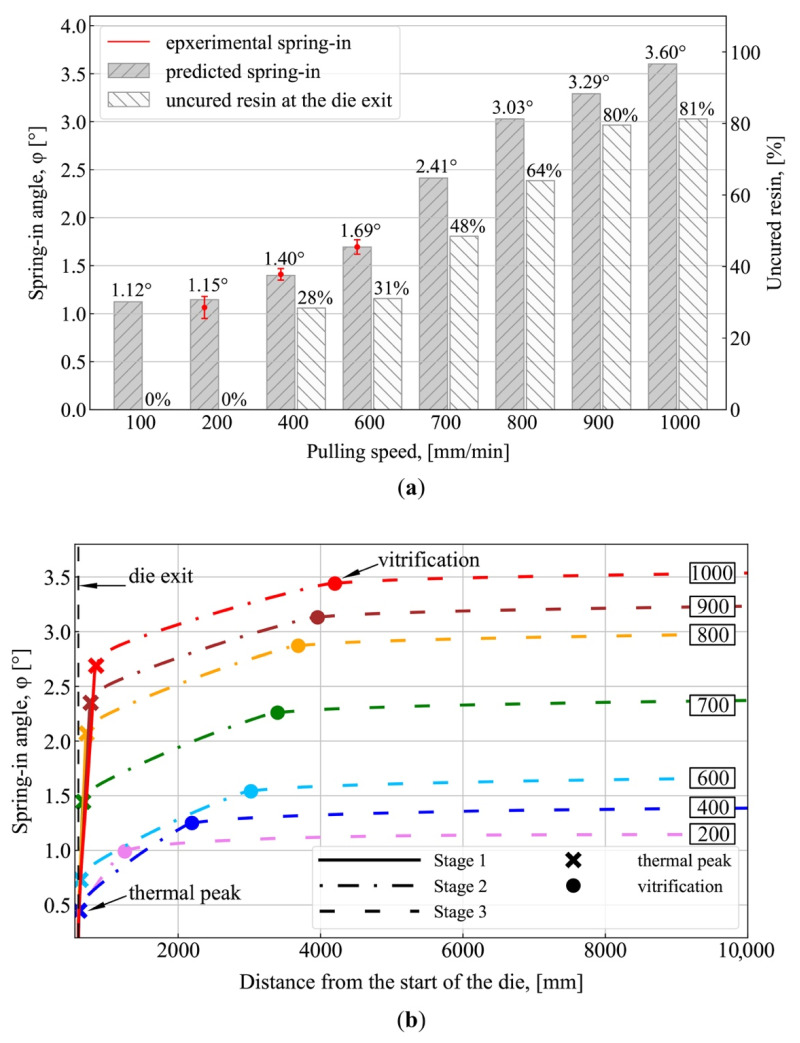
Numerical simulation results: (**a**) Final values of spring-in vs. fraction of uncured resin (α < 85%) at the die exit for different pulling speeds, obtained with the model described in “Modeling”; (**b**) Spring-in evolution during fabrication for different pulling speeds, obtained with the model described in “Modeling”. The solid lines in (**b**) correspond to Stage I (spring-in evolutions from the moment the profile exits the die block to the exothermic peak occurrence); the dot–dash line corresponds to Stage II (from the exothermic peak occurrence to the vitrification point); the dashed line corresponds to Stage III (after vitrification and to the full cooldown of the profile); the bold cross marks the occurrence of the exothermic peak; the bold point marks the vitrification point; the values shown in rectangles correspond to pulling speed (mm/min).

**Figure 6:**
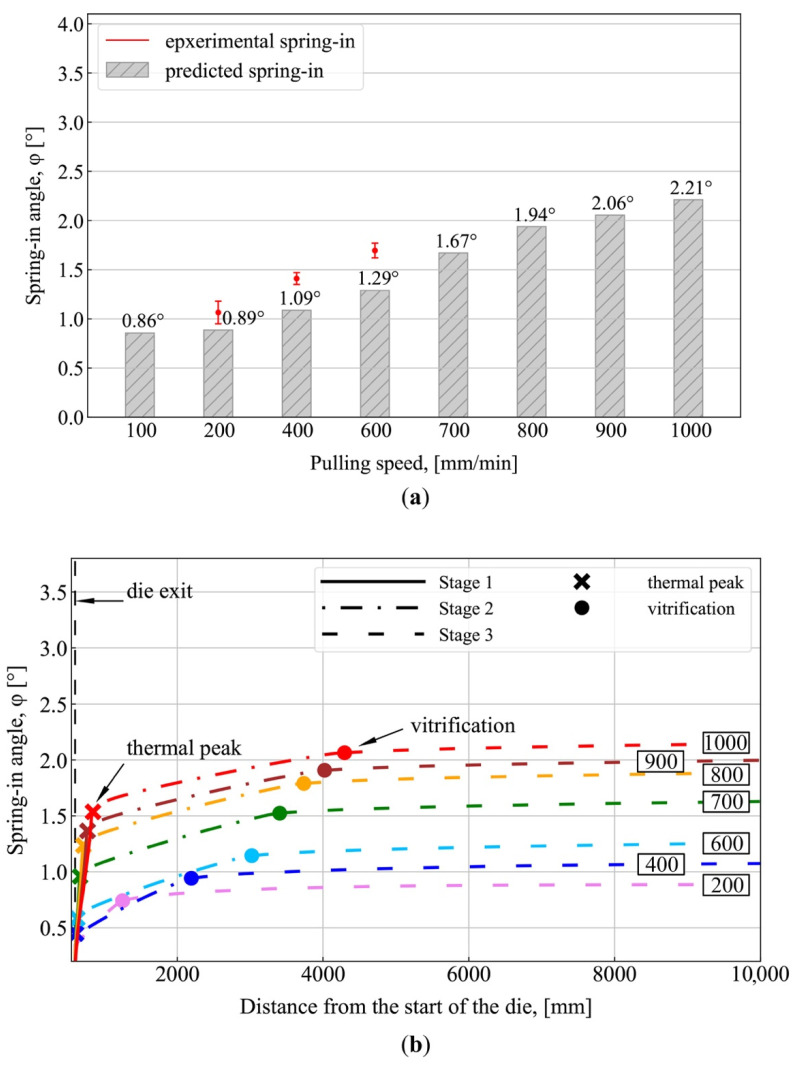
Numerical simulation results: (**a**) Final values of spring-in at the die exit for different pulling speeds, assuming the constant Poisson’s ratio of matrix; (**b**) Spring-in evolution during fabrication for different pulling speeds, assuming the constant Poisson’s ratio of matrix. The solid lines in (**b**) correspond to Stage I (spring-in evolutions from the moment the profile exits the die block and to the exothermic peak occurrence); the dot–dash line corresponds to Stage II (from the exothermic peak occurrence and to the vitrification point); the dashed line corresponds to Stage III (after vitrification and to the full cooldown of the profile); the bold cross marks the occurrence of the exothermic peak; the bold point marks the vitrification point; the values shown in rectangles correspond to pulling speed (mm/min).

**Figure 7:**
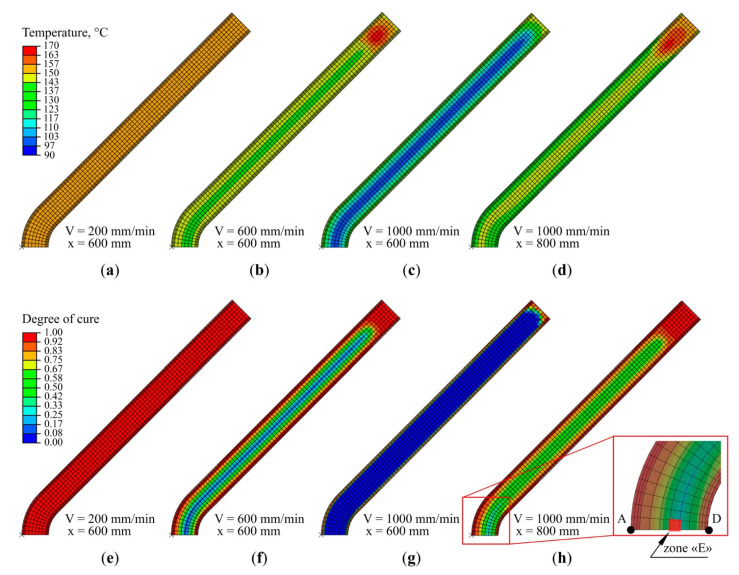
Simulation results: distribution of temperatures (**a**–**d**) and degree of polymerization (**e**–**h**) in the cross-section of the L-shaped profile: (**a**) pulling speed—200 mm/min, immediately after the die exit; (**b**) pulling speed—600 mm/min, immediately after the die exit; (**c**) 1000 mm/min, immediately after the die exit; (**d**) 1000 mm/min, at 200 mm after the die exit, or immediately after the post-die cooling tool; (**e**) 200 mm/min, immediately after the die exit; (**f**) 600 mm/min, immediately after the die exit; (**g**) 1000 mm/min, immediately after the die exit; (**h**) 1000 mm/min, at 200 mm after the die exit or immediately after the post-die cooling tool. Exothermic peak and vitrification were analyzed in Zone E shown to the right in (**h**).

Figure 5 and Figure 6 show the spring-in simulation results obtained at various pulling speeds from 100 to 1000 mm/min. Figure 7 shows the distributions of temperature and degree of polymerization obtained at pulling speeds of 200, 600, and 1000 mm/min. Figure 5 shows the simulation results obtained with the model described in Section 2. Figure 5a shows the final spring-in values obtained at different pulling speeds, together with the experimental values of spring-in for pulling speeds of 200, 400, and 600 mm/min. At these speeds, the predicted values fall between corresponding experimental data points (see Table 1). For the pulling speed of 200 mm/min, the predicted value constitutes 1.15° and is located between 0.97° (obtained in Experiment 1) and 1.16° (Experiment 2). For the pulling speed of 400 mm/min, the predicted value of 1.40° falls between 1.40° (Experiment 1) and 1.42° (Experiment 2). For the speed of 600 mm/min, the predicted value of 1.69° falls between the corresponding experimental values of 1.67° (Experiment 1) and 1.72° (Experiment 2). A slight decrease in spring-in values from 1.15° to 1.12° can be observed with the reduction in pulling speed from 200 to 100 mm/min. Starting from 200 mm/min, the increase in pulling speed results in a considerable increase in spring-in values. Thus, the increase in pulling speed from 200 to 1000 mm/min results in an over 3 times increase in spring-in (from 1.15° to 3.60°). Figure 5a also shows the fraction of uncured matrix material (α < 85%) within the cross-section of the profile after the die exit. It can be seen that the increase in the fraction of uncured material at the die exit corresponds to an increase in the final values of spring-in. Thus, at the pulling speed of 200 mm/min, the exothermic peak is located inside the die block, and the composite exits the die block fully cured (see Figure 5a and Figure 7e), giving the final spring-in value of 1.15°. The increase in pulling speed forces the exothermic peak further along the pultrusion line, beyond the die block exit. Thus, at pulling speeds of 600 mm/min and 1000 mm/min, the fraction of uncured resin within the cross-section of the profile constitutes 31% (see Figure 5a and Figure 7f) and 81% (see Figure 5a and Figure 7g), resulting in final spring-in values of 1.69° and 3.60°, respectively.

Figure 5b shows the diagrams of spring-in changes during fabrication for pulling speeds of 200 to 1000 mm/min. The diagram shows three zones corresponding to three stages of spring-in evolution, as follows: Stage I (solid line) corresponds to spring-in changes from the moment the profile exits the die block and to the moment of exothermic peak occurrence (marked by the bold cross); Stage II (dotted and dashed line) corresponds to spring-in changes from the moment of the exothermic peak and to the vitrification point (marked by the bold point); Stage III (dashed line) corresponds to spring-in changes after vitrification and to the full cooldown of the profile. The occurrence of the exothermic peak and vitrification was analyzed within Zone E located at a distance of 2.23 mm from the internal surface of the profile along the AD axis of symmetry (Figure 7h), as simulations show that the maximum temperature of the exothermic peak over the whole section of a composite is observed exactly in this zone at all pulling speeds. Table 1 presents the final values of spring-in, together with the contributions of each stage described above.

Starting from the pulling speed of 400 mm/min, the largest contribution to the increase in final spring-in comes from the spring-in occurring at Stage I. This can be attributed to the increase in fraction of uncured material in a composite exiting the die block. It leads to an increase in the corresponding total chemical shrinkage of material in the unconstrained environment. Thus, Stage I (2.72°) contributes 76% to the final spring-in value obtained at 1000 mm/min (3.60°). This can be viewed as the quantitative confirmation of the qualitative results reported by Baran et al. in [48]. It should be noted that at low pulling speeds, the exothermic peak occurs within the die block and the contribution of Stage I to spring-in evolutions in the post-die region is virtually zero (see the corresponding values for pulling speeds of 100 and 200 mm/min in Table 1). At Stage II, a slight decrease in spring-in can be observed with an increase in pulling speed, which is associated with the lower temperature of the exothermic peak. This phenomenon takes place when the exothermic peak occurs outside the die block. With an increase in pulling speed, the composite material stays in the die block for a shorter period of time and takes less heat from the die block. As the same material is considered, the amount of heat generated during the polymerization of the resin is constant and does not depend on the value of chosen pulling speed. Therefore, increasing the pulling speed, the amount of heat transmitted to the composite during pultrusion decreases, and, therefore, the temperature of the exothermic peak also decreases. The Stage III spring-in value virtually does not depend on the pulling speed and constitutes 0.15–0.16°. Thus, for a pulling speed of 1000 mm/min, contributions from Stages II and III to the final spring-in are 20% and 4%, respectively.

Figure 6a,b show the results of spring-in simulation for the pultruded L-shaped profile, assuming a constant Poisson’s ratio of the matrix, $\nu_{r}$ = 0.35. It can be seen that for a constant Poisson’s ratio, the spring-in values are lower than those obtained in simulations that account for changes in Poisson’s ratio in accordance with Equation (13). Thus, at the pulling speed of 100 mm/min, the spring-in obtained at a constant Poisson’s ratio constitutes 0.86°, while when varying Poisson’s ratio, the spring-in constitutes 1.12°, making the difference of 30%. At the pulling speed of 1000 mm/min, the spring-in constitutes 2.21° at a constant Poisson’s ratio and 3.60° at a varying Poisson’s ratio, making the difference of 63%. For simulations with a constant Poisson’s ratio, predicted final values of spring-in are lower than those obtained in the second experiment conducted at pulling speeds of 200, 400, and 600 mm/min by up to 30%, 30%, and 33%, respectively.

**Figure 8:**
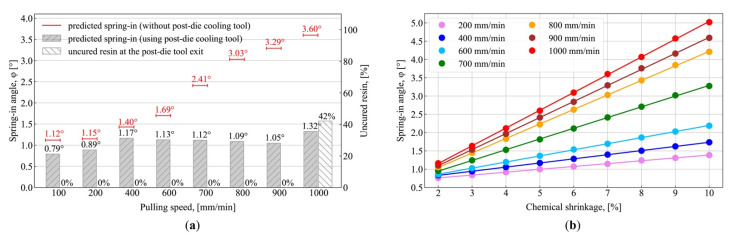
Simulation results for methods of spring-in reduction: (**a**) With the use of a post-die cooling tool. Gray columns mark the spring-in values obtained with the use of the post-die cooling tool; red lines indicate values obtained without the post-die cooling tool. The corresponding fraction of uncured resin (α < 85%) is shown at the bottom of the graph; (**b**) Spring-in vs. chemical shrinkage of the resin at different pulling speeds (for shrinkage values within the range of 2 to 10%).

Figure 8 shows the results of simulations conducted to estimate the efficiency of methods reducing the spring-in in composite parts, such as the use of a post-die cooling tool (Figure 8a), and the reduced chemical shrinkage of the matrix (Figure 8b). Figure 8a shows results of spring-in simulation in L-shaped profiles pultruded at different pulling speeds with the use of a rigid post-die cooling tool with a length constituting 1/3 of that of the die block ($L_{\mathrm{die}}/3$), which was installed in a pultrusion manufacturing line immediately after the exit of the heated die block.

To simulate the post-die cooling tool, boundary constraints within its region are set to prevent all motion at the outer perimeter of the profile (the ABCD segment in Figure 2c). The temperature conditions are set to simulate the cooling down of the profile after the die block exit according to Equation (4). After the exit from the post-die cooling tool, the boundary constraints are deactivated. As a result, a reduction in spring-in for all pulling speeds can be observed, as compared to the absence of the post-die cooling tool. The efficiency of the post-die cooling tool is more evident at higher pulling speeds. Thus, at low pulling speeds where the exothermic peak occurs within the die block, the reduction in spring-in constitutes 0.33° and 0.26° at 100 mm/min and 200 mm/min, respectively. However, at pulling speeds of 400–900 mm/min, where the exothermic peak shifts to the region of a post-die cooling tool, a significant reduction in the final value of spring-in can be observed, which is associated with the absence of uncured material in the profile exiting the post-die cooling tool (Figure 8a). Thus, at a pulling speed of 900 mm/min, the use of the post-die cooling tool results in a final spring-in value of 1.05°, which is 3.1 times lower than the final value of spring-in (3.29°) obtained at the same pulling speed but without the post-die cooling tool. A slight reduction in the final spring-in from 1.17° to 1.05° within the pulling speed range of 400–900 mm/min should also be noted, which can be attributed to the lower temperature of the exothermic peak; this affects the value of thermal shrinkage dependent on the temperature difference. With a further increase in pulling speed to 1000 mm/min, the exothermic peak shifts beyond the post-die cooling tool, resulting in the presence of uncured material at the exit of the post-die cooling tool and in chemical shrinkage taking place under unconstrained conditions. In turn, this results in the increased values of final spring-in compared to those obtained at pulling speeds of 400 to 900 mm/min. At the pulling speed of 1000 mm/min and with the use of the post-die cooling tool, the final value of spring-in is 1.32°, which is 2.7 times lower than the value of 3.60° obtained without the post-die cooling tool; this can be attributed to the lower fraction of uncured matrix material registered at the exit of the constrained environment of the post-die cooling tool, constituting 42% (see Figure 8a) versus 81% registered at the exit of the die block (see Figure 5a). Therefore, the use of the post-die cooling tool can be considered an effective technique to prevent the increase in spring-in at higher pulling speeds by reducing the fraction of uncured material in a composite exiting the constrained environment and, consequently, reducing the total chemical shrinkage occurring in the unconstrained environment.

Figure 8b shows the predictions of spring-in in L-shaped profiles pultruded at different pulling speeds for different values of chemical shrinkage in the range of 2 to 10%. A linear relationship can be observed between the chemical shrinkage of the matrix and the value of spring-in. Chemical shrinkage has a greater influence on the value of spring-in at higher pulling speeds. Thus, the final values of spring-in obtained at 1000 mm/min for 2% and 10% chemical shrinkage differ by a factor of 4.3, as compared to 1.8, which was obtained at a pulling speed of 200 mm/min. Therefore, the reduction of total chemical shrinkage is very important for resins with high chemical shrinkage as the difference between the final values of spring-in at different pulling speeds is particularly noticeable at higher values of chemical shrinkage.

## 4. Discussion

Spring-in formation in the post-die region takes place in three stages. Starting from the pulling speed of 400 mm/min, the largest contribution to the increase in final spring-in comes from Stage I located before the exothermic peak; this is associated with the exit of uncured resin from the die block and with the subsequent chemical shrinkage taking place in the unconstrained post-die region. The second contribution comes from Stage II, which takes place from the exothermic peak to the vitrification point. The lowest contribution to spring-in development comes from Stage III, which takes place from vitrification and to the complete cooldown of a composite. The increase in pulling speed raises the contribution from Stage I and reduces the role of Stage II, while the contribution from Stage III remains unchanged. Thus, for a pulling speed of 400 mm/min, the contributions to the final spring-in from Stages I, II, and III were 32% (0.45°), 57% (0.8°), and 11% (0.15°), respectively. At 1000 mm/min, the corresponding contributions constitute 76% (2.72°), 20% (0.72°), and 4% (0.16°), respectively.

To increase the efficiency and, thus, the profit from the pultrusion process, it is necessary to maximize pulling speed while preserving the quality of pultruded profiles. Simulations show that lower pulling speeds result in a reduction in spring-in. However, after a certain limit (see Figure 5a), a reduction in pulling speed does not produce a meaningful reduction in spring-in. Thus, the difference between the spring-in values obtained at pulling speeds of 100 and 200 mm/min was only 2.7%. Therefore, a reduction in pulling speed below the value corresponding to the exothermic peak location at the boundary between the die block and the unconstrained post-die region will only result in reduced process output and will not affect spring-in. At lower pulling speeds, the chemical shrinkage providing the largest contribution to spring-in development occurs within the die block. Here, a composite is contained in constrained conditions and, consequently, experiences less deformation compared to the unconstrained environment of the post-die region. Conversely, higher pulling speeds shift the exothermic peak beyond the constrained region of the die block, resulting in higher spring-in values. Thus, final spring-in values obtained at 200 mm/min (with an exothermic peak located inside the die block) and at 1000 mm/min (with an exothermic peak located in the post die region), differ by the factor of 3.1, constituting 1.15° and 3.60°, respectively.

Hence, to increase the process output, the capability is needed to reduce the contributions from chemical and thermal shrinkages to trade the slight increase in spring-in for a significant increase in pulling speed. The spring-in can be reduced by installing a post-die cooling tool or by using additives that reduce chemical shrinkage of the resin (carbon nanofibers [41], silica nanoparticles [103], aluminum oxide nanoparticles [78], and low-profile additives [104]). The use of a post-die cooling tool makes it possible to significantly increase the process output by increasing the pulling speed without increasing the final spring-in value. Thus, using the post-die cooling tool at the pulling speed of 900 mm/min makes it possible to obtain the same level of spring-in as at 200 mm/min without the cooling tool. Thus, the pulling speed can be increased by a factor of 4.5 while maintaining the same level of spring-in, i.e., 1.05° and 1.15°, respectively (see Figure 8a). The efficiency of this method can be explained by the smaller fraction of uncured material exiting the constrained environment. In turn, this results in a reduction in the total chemical shrinkage of a profile under unconstrained conditions. Thus, at 900 mm/min, the final value of spring-in obtained with the use of the post-die cooling tool is 1.05°, which is 3.1 times less than that obtained without the use of a cooling tool, where the final spring-in constitutes 3.29°.

Reduction of the total chemical shrinkage is also a very effective method of reducing the spring-in, which plays an important role in the case of resins featuring high chemical shrinkage because the difference between the final values of spring-in at different pulling speeds becomes more evident at higher values of chemical shrinkage. Thus, for resins with chemical shrinkage of 10%, the final values of spring-in obtained at pulling speeds of 200 mm/min and 1000 mm/min differ by a factor of 3.6, i.e., 1.39° and 5.02°, respectively. For resins with 2% chemical shrinkage, the corresponding values differ only by a factor of 1.5, i.e., 0.76° and 1.16°, respectively. In addition, at a pulling speed of 1000 mm/min, the use of additives to reduce the chemical shrinkage of resin from 7 to 2% makes it possible to obtain the level of spring-in equal to that of a resin with a chemical shrinkage of 7% without additives, at a pulling speed of 200 mm/min. That is, the pulling speed can be increased by as much as five times, maintaining constant spring-in values of 1.16° and 1.15°, respectively (see Figure 8b). The methods of reducing spring-in via the post-die cooling tool or with shrinkage-reducing additives require further investigation and experimental validation.

This 2D model is limited in that it does not account for changes in heat conduction in the pulling direction. However, it uses boundary conditions to account for the influence of pulling speed. The assumptions used in the model can lead to an overestimated exothermic peak as compared to the experimental values. In addition, the model does not account for stresses along the profile that can lead to additional shape deformations in the longitudinal direction. Nevertheless, these assumptions can be considered allowable in stress–strain analysis, considering large dimensions of produced profile in the pulling direction. In addition, these assumptions produce acceptable predictions of spring-in falling within spring-in values obtained in two pultrusion experiments. In future research, the authors intend to perform 3D analysis for the case of pultruded flat laminate to evaluate the influence of pulling speed and of profile thickness on the formation of cracks and distortions. Thus, a novel steady-state 3D-Eulerian numerical framework is intended to be applied in future works with the aim of accelerating the computational process [31].

The results of this study support findings in Baran et al. [48] that higher pulling speeds lead to increase in spring-in. However, their study is somewhat limited in that it analyzed only four different pulling speeds, and simulation results were experimentally validated only at one pulling speed. Consequently, the authors were unable to conclude that the decrease in spring-in observed with a reduction of pulling speed takes place only to a certain level, and that further reduction of pulling speed would not change the spring-in level. In their simulations, Baran et al. [48,49] assumed the constant and temperature-independent heat capacity of a composite. Considering that, according to our studies, heat capacity significantly affects the final value of spring-in, the assumption of constant and temperature-independent heat capacity of a composite appears unreasonable and will result in considerable disagreement between experimental results and predictions. Our study also demonstrate that chemical shrinkage plays a significant role in spring-in development in pultruded profiles, supporting the results reported by Wang et al. [70]. Moreover, the results obtained by Wang et al. are further extended in our study by calculating the contribution of thermal and chemical shrinkage to the growth of spring-in. The simulations demonstrated that reduced chemical shrinkage results in spring-in reduction, as shown in experiments with additives [41,78]. Moreover, the additives can also reduce the coefficient of thermal expansion of resin and, therefore, the spring-in angle, as shown in [81]. However, no experimental studies on the influence of additives on the development of cure-induced residual stresses and deformations in pultruded profiles have been published before. Therefore, this question will require further investigation. A significant reduction of cure-induced strains and spring-in can also be achieved through the use of microwave curing, as was demonstrated by Li et al. [47]. The influence of microwave processing on cure-induced strains in pultrusion also requires further investigation, as the application of this process seems to be quite possible [105]. Further experimental investigation of chemical shrinkage in pultrusion is intended to be performed with the use of Fiber Bragg Grating sensors as was done for autoclave technology by Hu et al. [77]. In addition, at high pulling speeds, it is necessary to consider the possible formation of matrix cracks and delamination, reducing the structural performance of pultruded profiles [60]. Therefore, further numerical studies are necessary to analyze the influence of the pultrusion process conditions on the formation of matrix cracks and delamination. It is also necessary to conduct multicriteria optimization of manufacturing conditions to maximize the pulling speed and minimize cure-induced residual stresses, spring-in, and formation of matrix cracks/delaminations, as was already done for other processes [73,106]. The effect of fiber volume fraction variability on the development of residual stresses and, therefore, on spring-in occurrence is to be investigated as well [30]. The authors also intend to simulate the formation of process-induced defects (spring-in, matrix cracks, delaminations) and their influence on the structural performance of other standard [107,108,109], curved [20], and new types of profiles designed using topology optimization methods [110].

## 5. Conclusions

To better understand the formation of spring-in in profiles manufactured at different pulling speeds, an experimental study and numerical simulation of 75 mm × 75 mm × 6 mm L-shaped profiles of glass fiber/vinyl ester resin has been conducted. The modified CHILE model accounting for changes in theYoung’s modulus and Poisson’s ratio during phase transitions was used for simulations. The occurrence of spring-in in L-shaped profiles manufactured at pulling speeds of 200, 400, and 600 mm/min was simulated. Then, the simulation results were compared with experimental values obtained in two pultrusion experiments conducted at the interval of 6 months. The predictions show good agreement with the experimental data. The validated model was also used to simulate the influence of pulling speed and of changes in the Poisson’s ratio of the matrix during phase transitions on the value of spring-in. Subsequently, the methods of reducing spring-in with the use of a post-die cooling tool and by reducing the chemical shrinkage of the resin were simulated. The following findings can be reported:

- The final value of spring-in depends on the position of the exothermic peak and vitrification point on the pultrusion line, relative to the end of the constrained region of the die, which, in turn, is determined by the selected pulling speed. The closer the exothermic peak position to the constrained region of the die, the lower the obtained spring-in values. Alternatively, higher spring-in values can be observed with the increase in the distance of the peak from the die exit because the composite can no longer sustain stresses from chemical shrinkage in the unconstrained environment of the post-die region. It was shown that higher pulling speeds result in a higher fraction of uncured material in a composite exiting the constrained environment of the die block. This leads to an increase in the total chemical shrinkage of the material under unconstrained conditions and, hence, results in increased values of spring-in;
- Starting from the pulling speed of 400 mm/min, the largest contribution to spring-in comes from the chemical shrinkage of the resin, which takes place before the exothermic peak (Stage I), and from thermal shrinkage taking place before vitrification of the composite (Stage II). However, at the cooling stage (Stage III), thermal shrinkage resulted only in a slight increase in spring-in. The higher pulling speeds increase the contribution from Stage I and reduce the role of Stage II, while the spring-in contribution from Stage III remains unchanged;
- The use of a post-die cooling tool or reduction of resin chemical shrinkage allows a minimum of 4.5 times increase in process output to be obtained while preserving the same level of spring-in.

## Figures and Tables

**Table 1:** Spring-in of L-shaped profiles pultruded at different pulling speeds (experimental results and predictions). Contributions of stages to the final spring-in value.

Pulling Speed [mm/min]		Spring-In Angle [°]				
	Experiment		Model			
	Experiment 1	Experiment 2	Stage I (From the Die Block Exit to the Exothermic Peak)	Stage II (From the Exothermic Peak to the Vitrification Point)	STAGE III (From Vitrification to the Full Cooldown of the Profile)	Final Value
100	–	–	0	0.97	0.15	1.12
200	0.97	1.16	0	0.99	0.16	1.15
400	1.40	1.42	0.45	0.80	0.15	1.40
600	1.67	1.72	0.76	0.78	0.15	1.69
700	–	–	1.46	0.80	0.15	2.41
800	–	–	2.10	0.77	0.16	3.03
900	–	–	2.38	0.75	0.16	3.29
1000	–	–	2.72	0.72	0.16	3.60

## Data Availability

The data presented in this study are available on request from the corresponding author.

## Supplementary Materials

The following are available online at https://www.mdpi.com/article/10.3390/polym13162748/s1, Supplementary materials: Calculation of composite properties for heat-transfer problem and composite effective mechanical properties.

## Notations

$\rho_{r}$	density of resin, kg/m^3^
$\rho_{f}$	density of fiber, kg/m^3^
$\rho_{\mathrm{comp}}$	density of composite, kg/m^3^
$k_{r}$	thermal conductivity of resin, W/(m·°C)
$k_{f\_\mathrm{trans}}$	thermal conductivity of the fiber in the transverse direction, W/(m·°C)
$k_{f\_\mathrm{long}}$	thermal conductivity of the fiber in the longitudinal direction, W/(m·°C)
$k_{\mathrm{comp}}$	thermal conductivity in the cross-sectional plane, W/(m·°C)
T	instantaneous temperature, °C
$C_{p\_r}\left(T\right)$	temperature-dependent heat capacity of resin, J/(kg·°C)
$C_{p\_f}$	heat capacity of the fiber, J/(kg·°C)
$C_{p\_\mathrm{comp}}\left(T\right)$	temperature-dependent heat capacity of a composite, J/(kg·°C)
t	time, sec
q	heat released due to the exothermic reaction in polymer matrix, kJ
x, y	coordinates of a cross-section of the composite profile, mm
z	coordinate of a composite cross-section along the pulling direction of pultrusion, mm
$h_{\mathrm{die}}$	convective heat transfer coefficient between the die block and the profile, W/(m^2^·°C)
$h_{\mathrm{air}}$	convective heat transfer coefficient between the ambient air and the profile after the die block exit, W/(m^2^·°C)
α	resin degree of cure
dα/dt	resin curing rate, 1/s
$A_{0}$	pre-exponential coefficient, 1/s
$E_{a}$	activation energy, kJ/mol
R	universal gas constant, J/(mol·°C)
n	order of reaction
$K_{\mathrm{cat}}$	activation constant
$H_{\mathrm{tot}}$	total heat released, kJ/kg
$T_{\mathrm{in}}$	temperature of material at the die block entrance, °C
$T_{\mathrm{die}}$	temperature at the die block, °C
$T_{1}$	temperature at the first zone of the die block, °C
$T_{2}$	temperature at the second zone of the die block, °C
$T_{3}$	temperature at the third zone of the die block, °C
$T_{4}$	temperature at the fourth zone of the die block, °C
$T_{5}$	temperature at the fifth zone of the die block, °C
$T_{6}$	temperature at the die block exit, °C
$T_{\mathrm{amb}}$	ambient temperature, °C
$T_{g}\left(\alpha \right)$	instantaneous glass transition temperature, °C
$T_{g0}$	glass transition temperature of uncured resin, °C
$T_{g\infty }$	glass transition temperature of fully cured resin, °C
$T^{*}$	difference between the instantaneous glass transition temperature and the instantaneous temperature of the resin, °C
$T_{c1}$, $T_{c2}$, $T_{c3}$, $T_{c4}$, $T_{c5}$, $T_{c6}$	critical temperatures, °C
$E_{r}$	instantaneous Young’s modulus of the resin, MPa
$E_{r}^{0}$	Young’s modulus of resin at $T_{c1}=-30.8\;{}^{\circ}C$, MPa
$E_{r}^{1}$	Young’s modulus of resin at $T_{c2}=0.7\;{}^{\circ}C$, MPa
$E_{r}^{2}$	Young’s modulus of resin at $T_{c3}=18.4\;{}^{\circ}C$, MPa
$E_{r}^{3}$	Young’s modulus of resin at $T_{c4}=30.5\;{}^{\circ}C$, MPa
$E_{r}^{4}$	Young’s modulus of resin at $T_{c5}=62.6\;{}^{\circ}C$, MPa
$E_{r}^{\infty }$	Young’s modulus of resin at $T_{c6}=95.1\;{}^{\circ}C$, MPa
$K_{r}\left(E_{r}\right)$	instantaneous bulk modulus of resin, MPa
$K_{r}^{\infty }$	bulk modulus of resin at T=25.3 °C, MPa
$K_{r}^{0}$	bulk modulus of resin at $T=T_{g\infty }$, MPa
$\nu_{r}\left(E_{r}\right)$	instantaneous Poisson’s ratio of resin
$\nu_{r}^{\infty }$	Poisson’s ratio of resin at T=25.3 °C
$\alpha_{r}^{\infty }$	coefficient of thermal expansion of resin at $T<T_{g\infty }$, 1/°C
$\alpha_{r}^{0}$	coefficient of thermal expansion of resin at $T\geq T_{g\infty }$, 1/°C
$E_{f}$	Young’s modulus of glass fiber reinforcement, MPa
$\nu_{f}$	Poisson’s ratio of glass fiber reinforcement
$\alpha_{f}$	coefficient of thermal expansion of glass fiber reinforcement, 1/°C
u	pulling speed, mm/min
$V_{f\_1}$	volume fraction of fabric layer reinforcement
$V_{f\_2}$	volume fraction of unidirectional layer reinforcement
ΔV	total volumetric chemical shrinkage, %
$\alpha_{\mathrm{gel}}$	resin cure degree corresponding to the gelation
λ	material constant in Equation (10)
$L_{\mathrm{die}}$	die block length, m
φ	spring-in angle, °
$t_{s}$	thickness of the strips, mm
$L_{w}$	size of the L-shaped profile legs, mm

## Appendix A

**Table A1:** Model parameters.

Property	Source	Symbol	Value			Unit
Heat transfer problem						
Density of resin	^– a^	$\rho_{r}$	1140			kg/m^3^
Density of fiber	[49]	$\rho_{f}$	2560			kg/m^3^
Thermal conductivity of resin	^– e^	$k_{r}$	0.178			W/(m·°C)
Thermal conductivity of fiber in the transverse direction	[49]	$k_{f\_\mathrm{trans}}$	1.04			W/(m·°C)
Thermal conductivity of fiber in the longitudinal direction	[49]	$k_{f\_\mathrm{long}}$	11.4			W/(m·°C)
Heat capacity of resin depending on the temperature	^– b^	$C_{p\_r}\left(T\right)$	5.1 × T + 1080			J/(kg·°C)
Heat capacity of fiber	[49]	$C_{p\_f}$	670			J/(kg·°C)
Convective heat transfer coefficient between the die block and the profile	^– f^	$h_{\mathrm{die}}$	5000			W/(m^2^·°C)
Convective heat transfer coefficient between the ambient air and the profile after the die block exit	^– f^	$h_{\mathrm{air}}$	9			W/(m^2^·°C)
Cure kinetics						
Pre-exponential coefficient	[90]	$A_{0}$	10^9.34^			1/s
Activation energy	[90]	$E_{a}$	93.3			kJ/mol
Universal gas constant	[90]	R	8.31			J/(mol·°C)
Order of reaction	[90]	n	1.91			-
Activation constant	[90]	$K_{\mathrm{cat}}$	10^2.73^			-
Total heat released	[90]	$H_{\mathrm{tot}}$	189			kJ/kg
Temperature conditions						
Temperature at the die block, $T_{\mathrm{die}}\left(z\right)$:			Pulling speed, mm/min			
			200	400	600	
@ 97–103 mm (1st zone)	^– a^	$T_{1}$	45	31	30	°C
@ 197–203 mm (2nd zone)	^– a^	$T_{2}$	62	42	40	°C
@ 297–303 mm (3rd zone)	^– a^	$T_{3}$	84	66	89	°C
@ 397–403 mm (4th zone)	^– a^	$T_{4}$	127	95	119	°C
@ 497–503 mm (5th zone)	^– a^	$T_{5}$	159	127	141	°C
@ 600 mm (at the die block exit)	^– a^	$T_{6}$	153	148	147	°C
Temperature of material at the die block entrance	^– a^	$T_{\mathrm{in}}$	18			°C
Ambient temperature	^– a^	$T_{\mathrm{amb}}$	18			°C
Mechanical properties of resin						
Young’s modulus at $T_{c1}=-30.8\;{}^{\circ}C$	^– c^	$E_{r}^{0}$	21			MPa
Young’s modulus at $T_{c2}=0.7\;{}^{\circ}C$	^– c^	$E_{r}^{1}$	86			MPa
Young’s modulus at $T_{c3}=18.4\;{}^{\circ}C$	^– c^	$E_{r}^{2}$	1961			MPa
Young’s modulus at $T_{c4}=30.5\;{}^{\circ}C$	^– c^	$E_{r}^{3}$	2473			MPa
Young’s modulus at $T_{c5}=62.6\;{}^{\circ}C$	^– c^	$E_{r}^{4}$	3083			MPa
Young’s modulus at $T_{c6}=95.1\;{}^{\circ}C$	^– c^	$E_{r}^{\infty }$	3421			MPa
Poisson’s ratio at T=25.3 °C	[111]	$\nu_{r}^{\infty }$	0.35			-
Bulk modulus at T=25.3 °C	$\frac{E_{r}^{\infty }}{3\left(1-2\nu_{r}^{\infty }\right)}$	$K_{r}^{\infty }$	3801			MPa
Bulk modulus at $T=T_{g\infty }$	Kr∞2.5 [56]	$K_{r}^{0}$	1520			MPa
Coefficient of thermal expansion at $T<T_{g\infty }$	^– d^	$\alpha_{r}^{\infty }$	60 × 10^−6^			1/°C
Coefficient of thermal expansion at $T\geq T_{g\infty }$	2.5 ×${\;\alpha }_{r}^{\infty }$ [56]	$\alpha_{r}^{0}$	150 × 10^−6^			1/°C
Mechanical properties of glass fiber reinforcement						
Young’s modulus	[57]	$E_{f}$	73 080			MPa
Poisson’s ratio	[57]	$\nu_{f}$	0.22			-
Coefficient of thermal expansion	[57]	$\alpha_{f}$	5.04 × 10^−6^			1/°C
Other properties						
Pulling speed	^– a^	u	200/400/600			mm/min
The volume fraction of reinforcement						
Fabric layer	^– a^	$V_{f\_1}$	0.5			-
UD layer	^– a^	$V_{f\_2}$	0.59			-
Total volumetric chemical shrinkage	[100]	ΔV	−7			%
Glass transition temperature of the uncured resin	[56]	$T_{g0}$	−41			°C
Glass transition temperature of the fully cured resin	^– c^	$T_{g\infty }$	120.4			°C
Resin cure degree corresponding to the gelation	^– f^	$\alpha_{\mathrm{gel}}$	0.6			-
Material constant in Equation (10)	[49]	λ	0.4			-
Die block length	^– a^	$L_{\mathrm{die}}$	0.6			m

^a^ Measured value in pultrusion experiments. ^b^ Determined from Differential Scanning Calorimetry (DSC) data. ^c^ Determined from Dynamic Mechanical Analysis (DMA) data. ^d^ Determined from Thermomechanical Analysis (TMA) data. ^e^ Determined from Laser Flash Analysis (LFA) data. ^f^ Assumed value.
