# Supplementary material for: Modeling Spring-In of L-Shaped Structural Profiles Pultruded at Different Pulling Speeds
Source: Polymers (Basel). 2021 Aug 16;13(16):2748. doi: 10.3390/polym13162748 (PMC8398191; doi:10.3390/polym13162748)
Supplement: Supplementary file 1 [file polymers-13-02748-s001.zip › polymers-1323189-supplementary.pdf]

polymers MDPI

Supplementary materials

Calculation of composite properties for heat-transfer problem

Composite density [1]***:***

$$w_f = \frac{V_f}{V_f + \left(\frac{\rho_r}{\rho_f}(1 - V_f)\right)} \tag{1}$$

$$\frac{1}{\rho_{comp}} = \frac{w_f}{\rho_f} + \frac{(1 - w_f)}{\rho_r} \tag{2}$$

Where w_f is fiber mass fraction, V_f is fibre volume fraction, ρ_r is density of resin, ρ_f is density of fibers, ρ_{comp} is density of composite.

Composite heat capacity [1]***:***

$$C_{p\_comp} = C_{p\_f} w_f + C_{p\_r}(1 - w_f) \tag{3}$$

Where C_{p_f} is heat capacity of fibers, C_{p_r} is heat capacity of resin.

Thermal conductivity of unidirectional composite in longitudinal and transversal direction [2]***:***

$$\frac{1}{k_{comp\_ud\_long}} = \frac{w_f}{k_{f\_long}} + \frac{(1 - w_f)}{k_r} \tag{4}$$

$$\frac{1}{k_{comp\_ud\_trans}} = \frac{w_f}{k_{f\_trans}} + \frac{(1 - w_f)}{k_r} \tag{5}$$

Where k_{f_long} and k_{f_trans} are thermal conductivities of fibers in longitudinal and transversal direction.

Calculation of composite effective mechanical properties

The relationships for the calculation of the composite effective mechanical properties are defined in accordance with Self-Consistent Field Micromechanics (SCFM) approach on the basis of fiber and resin mechanical properties [3], [4]:

E_{1f} is Young's modulus of fibre reinforcement in longitudinal direction, ν_{12f} is Poisson's ratio of fibre reinforcement in longitudinal direction, G_{12f} is shear modulus of fibre reinforcement in longitudinal direction, ν_{23f} is Poisson's ratio of fibre reinforcement in transversal direction, k_f is isotropic plane strain bulk modulus of fibre, V_F is fibre volume fraction.

E_r is instantaneous resin modulus, ν_r is instantaneous Poisson's ratio of resin, G_r instantaneous is shear modulus of resin, k_r is instantaneous isotropic plane strain bulk modulus of resin.

Young's modulus of composite in longitudinal direction:

$$E_1 = E_{1f}V_f + E_r(1-V_f) + \left[\frac{4(\nu_r - \nu_{12f}^2)k_f k_r G_r(1-V_f)V_f}{(k_f+G_r)k_r + (k_f-k_r)G_rV_f}\right] \tag{6}$$

Isotropic plane strain bulk modulus of fibre and resin:

$$k_f = \frac{E_{1f}}{2(1-\nu_{12f}-2\nu_{12f}^2)} \tag{7}$$

$$k_r = \frac{E_r}{2(1-\nu_r-2\nu_r^2)} \tag{8}$$

Shear modulus of composite:

$$G_{12} = G_{13} = G_r\left[\frac{(G_{12f}+G_r)+(G_{12f}-G_r)V_f}{(G_{12f}+G_r)-(G_{12f}-G_r)V_f}\right] \tag{9}$$

$$G_{23} = \frac{G_r[k_r(G_r+G_{23f})+2G_{23f}G_r+k_r(G_{23f}-G_r)V_f]}{k_r(G_r+G_{23f})+2G_{23f}G_r-(k_r+2G_r)(G_{23f}-G_r)V_f} \tag{10}$$

$$G_{23f} = \frac{E_{3f}}{2(1+\nu_{23f})} \tag{11}$$

Young's modulus of composite in transversal direction:

$$E_2 = E_3 = \frac{1}{(4k_T)^{-1}+(4G_{23})^{-1}+(\nu_{12}^2/E_1)} \tag{12}$$

Where k_T is the effective plane strain bulk modulus:

$$k_T = \frac{(k_f+G_r)k_r+(k_f-k_r)G_rV_f}{(k_f+G_r)-(k_f-k_r)V_f} \tag{13}$$

Poisson's ratios of composite:

$$\nu_{12} = \nu_{13} = \nu_{12f}V_f + \nu_r(1-V_f) + \left[\frac{(\nu_r-\nu_{12f})(k_r-k_f)G_r(1-V_f)V_f}{(k_f+G_r)k_r+(k_f-k_r)G_rV_f}\right] \tag{14}$$

$$\nu_{23} = \frac{2E_1k_T - E_1E_2 - 4\nu_{12}^2k_TE_2}{2E_1k_T} \tag{15}$$

Effective mechanical properties of quasi-isotropic laminate

The relationships for the calculation of the quasi-isotropic laminate effective mechanical properties are presented as in [4], [5], [6]. E_1, E_2 are Young's moduli of composite in longitudinal and transversal direction, G_{12}, G_{23} are the in-plane and out-of-plane shear moduli, ν_{12}, ν_{23} are the in-plane and out-of-plane Poisson's ratios. Mentioned properties are calculated as effective mechanical properties of unidirectional laminate by the Self-Consistent Field Micromechanics (SCFM) approach with the same volume fraction of reinforcement as the quasi-isotropic laminate. These properties are then used to calculate effective mechanical properties of quasi-isotropic laminate.

In-plane Young's modulus of composite:

$$E_x = E_y = 2\left(1 + \nu_{xy}\right)G_{xy} \tag{16}$$

In-plane Poisson's ratio of composite:

$$\nu_{xy} = \frac{-\frac{1}{2}G_{12} + \frac{1}{8}\frac{E_1(E_1 + E_2 + 6\nu_{12}E_2)}{E_1 - \nu_{12}^2}E_2}{\frac{1}{2}G_{12} + \frac{1}{8}\frac{E_1(3E_1 + 3E_2 + 2\nu_{12}E_2)}{E_1 - \nu_{12}^2}E_2} \tag{17}$$

In-plane shear modulus of composite:

$$G_{xy} = \frac{1}{2}G_{12} + \frac{1}{8}\frac{E_1(E_1 + E_2 - 2\nu_{12}E_2)}{E_1 - \nu_{12}^2E_2} \tag{18}$$

Out-of-plane Young's modulus of composite:

$$E_z = \frac{E_1 + (1 + 2\nu_{12})E_2}{(1 - \nu_{23}^2)\frac{E_1}{E_2} + (1 + 2\nu_{12} + 2\nu_{12}\nu_{23}) - \nu_{12}^2\frac{E_2}{E_1}} \tag{19}$$

Out-of-plane Poisson's ratio of composite:

$$\nu_{xz} = \nu_{yz} = \frac{E_x}{E_1}\frac{(\nu_{12} + \nu_{23} + \nu_{12}\nu_{23}) + \nu_{12}^2\frac{E_2}{E_1}}{1 + (1 + 2\nu_{12})\frac{E_2}{E_1}} \tag{20}$$

Out-of-plane shear modulus of composite:

$$G_{xz} = 2\left(\frac{G_{12}G_{23}}{G_{12} + G_{23}}\right) \tag{21}$$

Thermal strain

In-plane coefficient of thermal expansion of unidirectional laminate [3]*:*

$$\alpha_1 = \frac{\alpha_{1f}E_{1f}V_f + \alpha_r E_r(1 - V_f)}{E_{1f}V_f + E_r(1 - V_f)} \tag{22}$$

Where α_r is coefficient of thermal expansion of resin, α_{1f}, α_{2f} are the coefficients of thermal expansion of fibre reinforcement in longitudinal and transversal direction.

Out-of-plane coefficient of thermal expansion of unidirectional laminate [3]*:*

$$\alpha_2 = \alpha_3 = (\alpha_{2f} + \nu_{12f}\alpha_{1f})V_f + \alpha_r(1 + \nu_r)(1 - V_f) - \left(\nu_{12f}V_f + \nu_r(1 - V_f)\right)\alpha_1 \tag{23}$$

In-plane coefficient of thermal expansion of quasi-isotropic laminate [7]***:***

$$\alpha_x = \alpha_y = \frac{(E_1 + \nu_{12}E_2)\alpha_1 + (1 + \nu_{12})E_2\alpha_2}{E_1 + (1 + 2\nu_{12})E_2} \tag{24}$$

Out-of-plane coefficient of thermal expansion of quasi-isotropic laminate [7]***:***

$$\alpha_z = \frac{(\nu_{12}E_2 - \nu_{23}E_1)\alpha_1 + \big((1 + \nu_{23})E_1 + (1 + \nu_{12})E_2\big)\alpha_2}{E_1 + (1 + 2\nu_{12})E_2} \tag{25}$$

Incremental effective thermal strain of the composite:

$$\Delta\varepsilon_i^{th} = \alpha_i \cdot \Delta T \tag{26}$$

Chemical strain

Incremental specific volume shrinkage of the resin [3]***:***

$$\Delta V_r = \Delta\alpha \cdot V_{sh} \tag{27}$$

Where $\Delta\alpha$ is change in the degree of cure, V_{sh} is total volumetric resin shrinkage

Isotropic incremental resin shrinkage strain [3]***:***

$$\Delta\varepsilon_r = \Delta V_r/3 \tag{28}$$

Effective in-plane incremental chemical shrinkage strain of unidirectional laminate [3]***:***

$$\Delta\varepsilon_1^{ch} = \frac{\Delta\varepsilon_r E_r(1 - V_f)}{E_{1f}V_f + E_r(1 - V_f)} \tag{29}$$

Effective out-of-plane incremental chemical shrinkage strain of unidirectional laminate [3]***:***

$$\Delta\varepsilon_2^{ch} = \Delta\varepsilon_3^{ch} = \Delta\varepsilon_r(1 + \nu_r)(1 - V_f) - \big(\nu_{12f}V_f + \nu_r(1 - V_f)\big)\ \Delta\varepsilon_1^{ch} \tag{30}$$

Effective in-plane incremental chemical shrinkage strain of quasi-isotropic laminate [7]***:***

$$\Delta\varepsilon_x^{ch} = \Delta\varepsilon_y^{ch} = \frac{(E_1 + \nu_{12}E_2)\Delta\varepsilon_1^{ch} + (1 + \nu_{12})E_2\Delta\varepsilon_2^{ch}}{E_1 + (1 + 2\nu_{12})E_2} \tag{31}$$

Effective out-of-plane incremental chemical shrinkage strain of quasi-isotropic laminate [7]***:***

$$\Delta\varepsilon_z^{ch} = \frac{(\nu_{12}E_2 - \nu_{23}E_1)\Delta\varepsilon_1^{ch} + \big((1 + \nu_{23})E_1 + (1 + \nu_{12})E_2\big)\Delta\varepsilon_2^{ch}}{E_1 + (1 + 2\nu_{12})E_2} \tag{32}$$

Stress-strain analysis

Effective mechanical properties of composite, obtained previously, are then used for the calculation of Jacobian matrix (**J**). Subsequently, in the case of orthotropic material, the incremental stress tensor ($\Delta\sigma_{ij}$) is calculated based on the Jacobian matrix (**J**) and incremental mechanical strain tensor ($\Delta\varepsilon_{ij}^{mech}$) [3], [8]. At the end of each increment the update of the stress and strains tensors is taking place.

Total incremental strain:

$$\Delta\varepsilon_{ij}^{tot} = \Delta\varepsilon_{ij}^{mech} + \Delta\varepsilon_{ij}^{th} + \Delta\varepsilon_{ij}^{ch} \tag{33}$$

Incremental process induced strain:

$$\Delta\varepsilon_{ij}^{pr} = \Delta\varepsilon_{ij}^{th} + \Delta\varepsilon_{ij}^{ch} \tag{34}$$

Incremental mechanical strain:

$$\Delta\varepsilon_{ij}^{mech} = \Delta\varepsilon_{ij}^{tot} - \Delta\varepsilon_{ij}^{pr} \tag{35}$$

Incremental stress tensor:

$$\Delta\sigma_{ij} = \mathbf{J}\Delta\varepsilon_{ij}^{mech} \tag{36}$$

Update at the end of each increment:

$$\varepsilon_{ij}^{n+1} = \varepsilon_{ij}^{n} + \Delta\varepsilon_{ij}^{n} \tag{37}$$

$$\sigma_{ij}^{n+1} = \sigma_{ij}^{n} + \Delta\sigma_{ij}^{n} \tag{38}$$

Table S1. Material properties defined in ABAQUS

Property	*Symbol*	*Value*	*Unit*
	Material_1		
Volume fraction of reinforcement	V_f	*0.59*	-
Mass fraction of reinforcement	w_f	*0.764*	-
Mass fraction of resin	w_r	*0.236*	-
Axis of the reinforcement (longitudinal)	-	*3*	-
Thermal conductivity in the longitudinal direction	$k_{comp\_ud\_long}$	*0.717*	*W/(m·ºC)*
Thermal conductivity in the transversal direction	$k_{comp\_ud\_trans}$	*0.485*	*W/(m·ºC)*
Density	ρ_{comp}	*1980*	*kg/m³*
Heat capacity	$C_{p\_comp}(T)$	*1.2·T+767*	*J/(kg·ºC)*
	Material_2		

Volume fraction of reinforcement	V_f	*0.5*	-
Mass fraction of reinforcement	w_f	*0.692*	-
Mass fraction of resin	w_r	*0.308*	-
Axis of the reinforcement (longitudinal)	-	*3*	-
Thermal conductivity in the longitudinal direction	$k_{comp\_ud\_long}$	*0.558*	*W/(m·ºC)*
Thermal conductivity in the transversal direction	$k_{comp\_ud\_trans}$	*0.417*	*W/(m·ºC)*
Density	ρ_{comp}	*1850*	*kg/m³*
Heat capacity	$C_{p\_comp}(T)$	*1.57·T+796*	*J/(kg·ºC)*
		Material_3	
Volume fraction of reinforcement	V_f	*0.5*	-
Mass fraction of reinforcement	w_f	*0.692*	-
Mass fraction of resin	w_r	*0.308*	-
Axis of the reinforcement (longitudinal)	-	*1*	-
Thermal conductivity in the longitudinal direction	$k_{comp\_ud\_long}$	*0.558*	*W/(m·ºC)*
Thermal conductivity in the transversal direction	$k_{comp\_ud\_trans}$	*0.417*	*W/(m·ºC)*
Density	ρ_{comp}	*1850*	*kg/m³*
Heat capacity	$C_{p\_comp}(T)$	*1.57·T+796*	*J/(kg·ºC)*
		Material_4	
Volume fraction of reinforcement	V_f	*0.5*	-
Mass fraction of reinforcement	w_f	*0.692*	-
Mass fraction of resin	w_r	*0.308*	-
Lay-up plane	-	*1-3*	-
Thermal conductivity in the lay-up plane	$k_{comp\_fabric\_0}$	*0.732*	*W/(m·ºC)*
Thermal conductivity perpendicular to the lay-up plane	$k_{comp\_fabric\_90}$	*0.559*	*W/(m·ºC)*

Density	ρ_{comp}	*1850*	*kg/m³*
Heat capacity	$C_{p\_comp}(T)$	*1.57·T+796*	*J/(kg·ºC)*

(1) Batch, G. L.; Macosko, C. W. Heat Transfer and Cure in Pultrusion: Model and Experimental Verification. *AIChE J.* **1993**, *39* (7), 1228–1241. https://doi.org/10.1002/aic.690390713.

(2) Chachad, Y. R.; Roux, J. A.; Vaughan, J. G.; Arafat, E. Three-Dimensional Characterization of Pultruded Fiberglass-Epoxy Composite Materials. *J. Reinf. Plast. Compos.* **1995**, *14* (5), 495–512. https://doi.org/10.1177/073168449501400506.

(3) Bogetti, T. A.; Gillespie J.W., J. Process-Induced Stress and Deformation in Thick-Section Thermoset Composite Laminates. *J. Compos. Mater.* **1992**, *26* (5), 626–660. https://doi.org/10.1177/002199839202600502.

(4) Baran, I. Modelling the Pultrusion Process of Off Shore Wind Turbine Blades, Technical University of Denmark, 2014.

(5) Tsai, S. W.; Hahn, H. T. *Introduction to Composite Materials*; New York, 1980. https://doi.org/https://doi.org/10.1201/9780203750148.

(6) Goetschel, D. B.; Radford, D. W. Analytical Development of Through-Thickness Properties of Composite Laminates. *J. Adv. Mater.* **1997**, *28* (4), 37–46.

(7) Akkerman, R. On the Properties of Quasi-Isotropic Laminates. *Compos. Part BEngineering* **2002**, *33* (2), 133–140. https://doi.org/10.1016/S1359-8368(02)00002-1.

(8) Johnston, A.; Vaziri, R.; Poursartip, A. A Plane Strain Model for Process-Induced Deformation of Laminated Composite Structures. *J. Compos. Mater.* **2001**, *35* (16), 1435–1469. https://doi.org/10.1106/YXEA-5MH9-76J5-BACK.
